# T17. OXIDATIVE STRESS BIOMARKERS AND NEGATIVE DIMENSION IN THE FIRST TEN YEARS OF SCHIZOPHRENIA: A 1-YEAR FOLLOW-UP STUDY

**DOI:** 10.1093/schbul/sby016.293

**Published:** 2018-04-01

**Authors:** Leticia González-Blanco, M Paz Garcia-Portilla, Leticia Garcia-Alvarez, Lorena De La Fuente-Tomas, Pilar Saiz-Martinez, Celso Iglesias, Ana Coto, Julio Bobes

**Affiliations:** University of Oviedo

## Abstract

**Background:**

Several studies have documented changes in oxidative parameters and antioxidant enzymes in patients with schizophrenia (1, 2). However, their relation to negative symptoms and the longitudinal clinical course is still unclear.

The objectives of the present study are to: 1) analyze the association between oxidative stress biomarkers and negative dimension; 2) identify if these biomarkers could predict clinical outcomes in stable patients with schizophrenia at 1-year follow-up.

**Methods:**

A 1-year follow-up study of 57 stable outpatients with schizophrenia (≤10 years of illness) (mean age=31.5 ± 6.5; 63.2% males).

**Assessment:**

PANSS, Clinical Assessment Interview of Negative Symptoms (CAINS) -Motivation/Pleasure (MAP) & Expression (EXP) domains-, Brief Negative Symptom Scale (BNSS). Oxidative stress biomarkers: homocysteine, hemolysis test (% hemolysis), lipid peroxidation subproducts (LPO), catalase activity in erythrocytes (CAT).

Pearson correlations were performed to determine associations between biomarkers and clinical scores at baseline, and they were included in stepwise multiple linear regression analyses, considering potential confounding factors.

The clinical course for each psychopathological domain was determined using the formula: [follow-up-baseline scores]. Positive values were interpreted as worsening, while negative improvement. Pearson correlation and multiple linear regression analyses were performed to determine if baseline levels of oxidative stress parameters were predictors of clinical changes at follow-up.

**Results:**

1) Baseline associations: Final regression models identified that LPO level was a significant predictor of lower scores in PANSS-N, BNSS total, Avolition and Blunted Affect subscale of BNSS and CAINS-EXP (β= -0.408; -0.290, -0.254, -0.296, -0.247, respectively).

2) Longitudinal course: At 1-year follow-up, patients only improved significantly (p<0.05) in PANSS-Total [59.4 ± 16.4 - 54.5 ± 16.0 (t=3.362)], PANSS-General [29.7 ± 8.9 - 26.9 ± 7.9 (t=3.362)], Blunted Affect subscale [6.9 ± 5.0 - 5.9 ± 4.7 (t=2.489)], and almost significant (p<0.069) in CAINS-EXP and BNSS total score. No significant changes in BMI, waist circumference, smoking or antipsychotic equivalent doses were detected, but they were also considered in regression analyses. A higher percentage of hemolysis at baseline, with a decrease in equivalent doses of antipsychotics, both significantly predict an improvement in scores of PANSS-N (R2=0.140, F=7.166), BNSS (R2=0.246, F=6.193) and CAINS-EXP (R2=0.186, F=5.259).

**Discussion:**

Lower concentrations of LPO were related to greater severity of negative symptoms as avolition and blunted affect (inner world). Longitudinal analyses showed that higher % of hemolysis at baseline predict an improvement of negative dimension at 1-year follow-up. From our results, we hypothesize that there is an inverse relationship between oxidative stress and negative dimension in stable patients with schizophrenia during the first ten years of illness.

